# NFAT as a Biomarker and Therapeutic Target in Non–Small Cell Lung Cancer–Related Brain Metastasis

**DOI:** 10.3389/fonc.2021.781150

**Published:** 2021-11-30

**Authors:** Lu Peng, Yihao Tao, Rui Wu, Jing Su, Maoyuan Sun, Yuan Cheng, Zongyi Xie, Jinning Mao, Xiaohui Zhan, Guodong Liu

**Affiliations:** ^1^ Department of Neurosurgery, The Second Affiliated Hospital of Chongqing Medical University, Chongqing, China; ^2^ Department of Cardiology, The Second Affiliated Hospital of Chongqing Medical University, Chongqing, China; ^3^ Department of Bioinformatics, School of Basic Medical Sciences of Chongqing Medical University, Chongqing, China

**Keywords:** non–small cell lung cancer, brain metastasis, biomarker, bioinformatics, immunohistochemistry

## Abstract

**Background:**

Brain metastases (BMs) are associated with poor prognosis and significant mortality, and approximately 25% of patients with non–small cell lung cancer (NSCLC) develop BMs. The present study was aimed to understand the relationships between BM and NSCLC and reveal potential biomarkers and therapeutic targets in NSCLC-related BM.

**Methods:**

The differentially expressed genes (DEGs) expressed during NSCLC and BM development were predicted by bioinformatics analysis, and the expression of the upstream transcription factor nuclear factor of activated T cells (NFAT) was confirmed as a differential gene expressed in both NSCLC and BM. In addition, the expression of proteins encoded by these DEGs was verified by immunohistochemical experiments examining normal lung tissue, lung cancer tissue, and brain metastasis tissue from 30 patients with NSCLC related BM.

**Results:**

The co-DEGs interleukin (IL)-11, cadherin 5 (CDH5) and C-C motif chemokine 2 (CCL2) link NSCLC and BM in the Gene Expression Omnibus (GEO) database, and NFAT may target the expression of these co-DEGs. In the GEO database, NFATc1 and NFATc3 were significantly downregulated in NSCLC tissues (P <.05), whereas NFATc1, NFATc2, NFATc3, and NFATc4 were significantly downregulated in BMs (P <.05). Consistent findings were observed in the immunohistochemical analysis.

**Conclusion:**

NFATc1 and NFATc3 may play important roles in the occurrence of NSCLC and BM by regulating IL-11, CDH5, and CCL2.

## Introduction

Brain metastases (BMs) are associated with poor prognosis and significant mortality, and they are the most common central nervous system malignancies, developing in 20–40% of advanced-stage cancers; 40–50% of these cases occur in patients with lung cancer (1). Moreover, approximately 25% of patients with non-small cell lung cancer (NSCLC) develop BMs (2), and NSCLC accounts for about 88% of primary lung malignancies (3, 4). BM negatively impacts survival (5) and causes significant neurologic, cognitive, and emotional difficulties (6). Prognostic factors that improve overall survival include Karnofsky performance status score >70 (7, 8), age <65 years, controlled primary tumor, and no extracranial metastases (9).

Efforts to characterize patients who develop BM have been fairly disappointing. Prophylactic cranial irradiation (PCI) is offered to small cell lung cancer patients with early stage disease that is stable after initial systemic treatment or has responded to therapy (10). However, if NSCLC patients who are likely to develop BM could be predicted and offered PCI (sparing the NSCLC patients who are unlikely to develop BM from PCI-related side effects), this may be useful. Although recent studies on the genomic etiology of NSCLC have enabled patients to be treated with individualized therapies, therapeutic options for BM remain limited because of inefficient drug transport across the blood–brain barrier (11). Furthermore, compared to matched primary tumors, BMs tend to harbor unique driver mutations (12, 13). Identifying key genomic signatures and the molecular mechanisms underlying BM development could allow novel therapeutic options to be identified. However, the molecular mechanisms of BMs related to lung cancer have not been extensively studied because of the invasiveness and difficulty of obtaining BM samples.

Bioinformatics analysis, especially differentially expressed gene (DEG) analysis and identification of the associated biological processes and pathways, has been used to reveal potential disease biomarkers. In this study, we elucidated the NSCLC-related DEGs (NSCLC-DEGs) and BM-related DEGs (BM-DEGs), and the co-differentially expressed genes (co-DEGs) that were shared between the NSCLC-DEGs and BM-DEGs. In addition, we conducted a bioinformatics analysis of the co-DEGs, elucidating the molecular mechanisms and predicting the regulator that may target the co-DEGs. Finally, we verified our results through immunohistochemistry experiments, thereby identifying potential biomarkers in NSCLC patients prone to BM.

## Methods

### Datasets

GSE161116 (13 lung tumor tissues and 15 brain tissues from 17 primary NSCLC patients with BM; 4 and 2 cases only provided brain and lung tissues, respectively), GSE74706 (18 lung tumor tissues and 18 corresponding normal tissues from primary NSCLC patients), and GSE21933 (21 lung tumor tissues and 21 corresponding normal tissues from primary NSCLC patients) were downloaded from the Gene Expression Omnibus (GEO) database (http://www.ncbi.nlm.nih.gov/geo/) (14). **Table 1** shows a detailed overview of the patient characteristics, including staging. The three datasets were used to identify DEGs and the molecular mechanisms underlying primary NSCLC and BM.

**Table 1:** Clinical characteristics of patients.

Characteristic	Lung tumor tissues		Brain metastases tissues
	BM (+)	BM (-)	
Age			
≤60	7	7	9
>60	6	32	6
Gender			
Male	8	28	8
Female	5	11	7
Grade			
IA or IB	–	12	–
IIA or IIB	–	7	–
IIIA or IIIB	–	14	–
IV	11	6	–
Histology			
ADC	9	21	9
SCC	2	18	2
Other NSCLC	2		4
Overall	13	39	15

ADC, adenocarcinoma; SCC, squamous cell carcinoma.

### Data Processing

Using the platform information related to the GPL19965, GPL13497, and GPL6254 platforms, the gene IDs were mapped to gene symbols. DEG analysis (fold change >2 and p<0.01) was performed for GSE161116, GSE21933 and GSE74706 RAW datasets using the R package “limma”. In addition, the top four GO biological process (BP) terms for the DEGs in each of the three datasets were determined using the DAVID Gene Functional Classification Tool (https://david.ncifcrf.gov/home.jsp) (15), and heatmaps of the expression of the DEGs in relation to each GO BP term were constructed. Additionally, we constructed a Venn diagram of NSCLC-DEGs (lung tumor tissues vs. brain tissues from primary NSCLC patients with BM) and BM-DEGs (lung tumor tissues vs. normal tissues from primary NSCLC patients), showing the shared DEGs (co-DEGs).

### Functional Enrichment Analyses

Gene Ontology (GO) and Kyoto Encyclopedia of Genes and Genomes (KEGG) enrichment analyses of BM- and NSCLC-DEGs were carried out using the R package “clusterProfiler”. GO biological process (BP), molecular function (MF), and cellular component (CC) terms and KEGG pathways with p<0.05 were considered to be significantly enriched. In addition, to verify the accuracy of the co-DEGs annotation, the AmiGO database (http://amigo.geneontology.org/amigo/) was used to confirm GO term enrichment related to the co-DEGs.

### Protein–Protein Interaction (PPI) Networks of DEGs

PPI networks of BM- and NSCLC-DEGs were constructed using Search Tool for the Retrieval of Interacting Genes/Proteins (STRING) v10.5 (http://string-db.org/), which predicts functional protein associations and PPIs. The analytic results with confidence scores >0.9 were then downloaded from STRING (16) to create the PPI networks. Proteins in the central nodes might have important physiological regulatory functions and might be key candidate genes. Subsequently, the genes in the most significant module were extracted and subjected to GO and KEGG pathway enrichment analysis at a significance of P < 0.05.

### Identification of Transcription Factors That Regulate co-DEGs

ToppGene Suite (https://toppgene.cchmc.org/), which is a one-stop portal for gene list enrichment analysis and candidate gene prioritization, based on functional annotations and protein interaction networks, was used (17). We determined potential transcription factors (TFs) by identifying the transcription factor-binding site (TFBS) in the top 20 co-DEGs based on p-value ≤ 0.05, according to prediction tools. The TFs associated with DEGs in all three datasets were identified.

### Survival Analysis of the co-DEGs

Survival analyses for the co-DEGs regulated by the identified TFs were performed by Gene Expression Profiling Interactive Analysis (GEPIA) (http://gepia2.cancer-pku.cn/), a valuable and highly cited resource for gene expression analysis based on tumor and normal samples from The Cancer Genome Atlas (TCGA) and the Genotype–Tissue Expression (GTEx) databases (18).

### Immunohistochemistry

We retrospectively collected BMs, lung cancer tissues, and matched neighboring normal lung tissue from 30 patients with BMs due to NSCLC who underwent surgical resection at the Second Affiliated Hospital of Chongqing Medical University, China (September 2016 to June 2021). Detailed clinical information was collected for each patient, including age, sex, tumor location, histological differentiation, and tumor lymph node metastasis (TNM) staging. The research protocol was approved by the Ethics Committee of Chongqing Medical University, and both clinicians and patients agreed to the use of collected tissues for research purposes.

Protein expression was immunohistochemically determined. Human anti-NFATc1 and anti-NFATc3 were purchased from Cell Signaling Technology (Danvers, MA, USA). In brief, paraffinized tissues were cut into 8-µm-thick sections, deparaffinized with xylene, and rehydrated in reduced concentrations of ethanol. Antigen retrieval was achieved by boiling the slices in 10 mM citrate buffer for 20 minutes. After blocking endogenous peroxidase with 3% catalase in methanol, the sections were incubated with the background sniper at room temperature for 30 minutes, followed by incubation with primary antibody at a working concentration of 1:100 at 4°C overnight then incubated with HRP-conjugated secondary antibodies. The sections were examined under a microscope (Olympus).

### Statistical Analysis

Based on our gene-expression data, the expression of NFAT was stratified into two categories: NFATlow or NFAThigh. Samples scored as NFATlow fell into two staining patterns: (A) complete lack of NFAT expression (Score 0); (B) scattered and faint cytoplasm expression in a minority fraction of cells (Score +). Samples scored as NFAThigh also fell into two staining patterns: (C) strong staining in a majority fraction of cells (Score ++); (D) strong staining in all cells (Score +++). For each sample, two independent tissue cores from distinct areas of the same lesion were analyzed. Tumors with discordant scores on the two cores were upgraded to the highest score. Two independent investigators used the same criteria (**Additional File 1**). The concordance between the two observers was analyzed using contingency tables to calculate the Cohen’s Kappa Index (**Additional Files 2**, **3**). Then, Chi-square test were used to make inter-group comparison. All statistical analyses were performed with SPSS (version 16.0) and GraphPad (Version 5.0).

## Results

### Identification of DEGs

**Figure 1:**
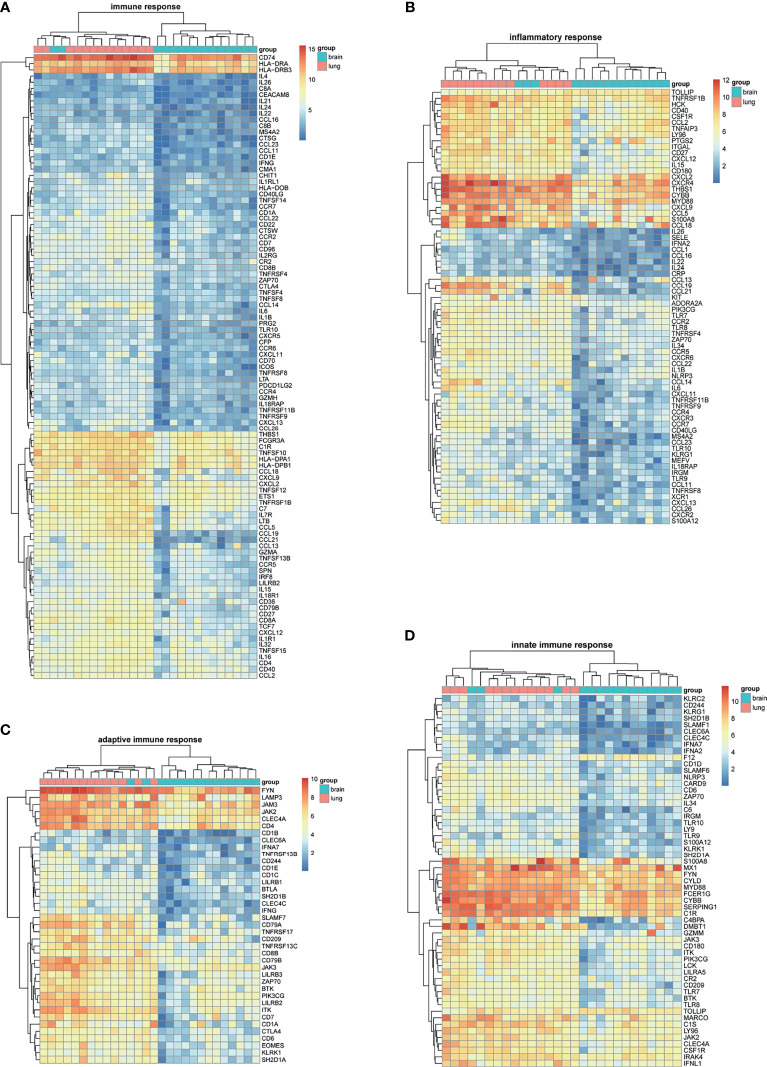
Heatmaps of BM-related differentially expressed genes (DEGs) based on GSE161116. **(A–D)** Hierarchical clustering heatmaps of DEG expression related to immune response, inflammatory response, adaptive immune response, and innate immune response.

**Figure 2:**
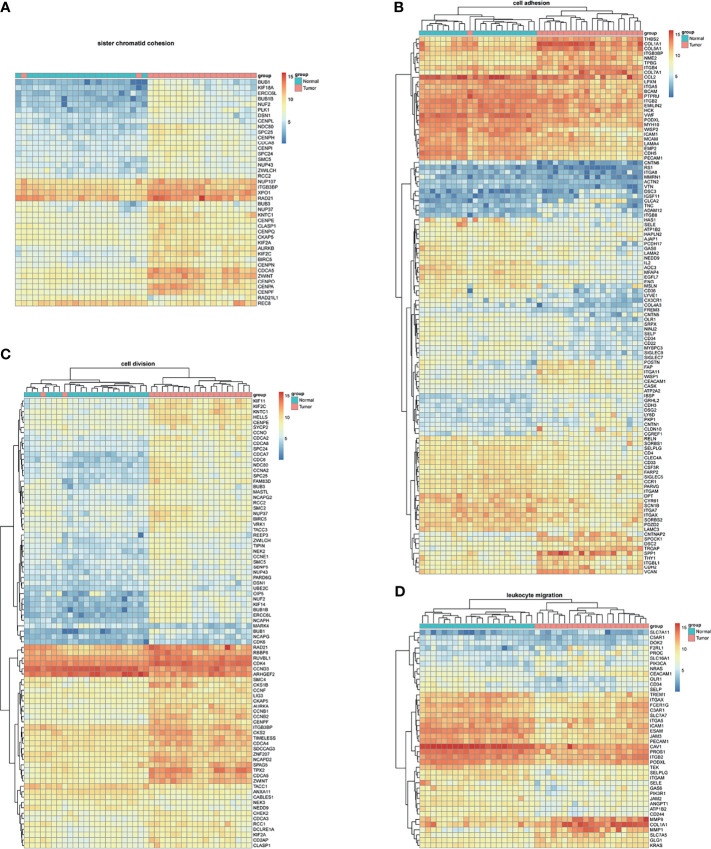
Heatmaps of NSCLC-related differentially expressed genes (DEGs) based on GSE74706. **(A–D)** Hierarchical clustering heatmaps of DEG expression related to mitotic nuclear division, epidermis development, DNA replication, and cell division.

**Figure 3:**
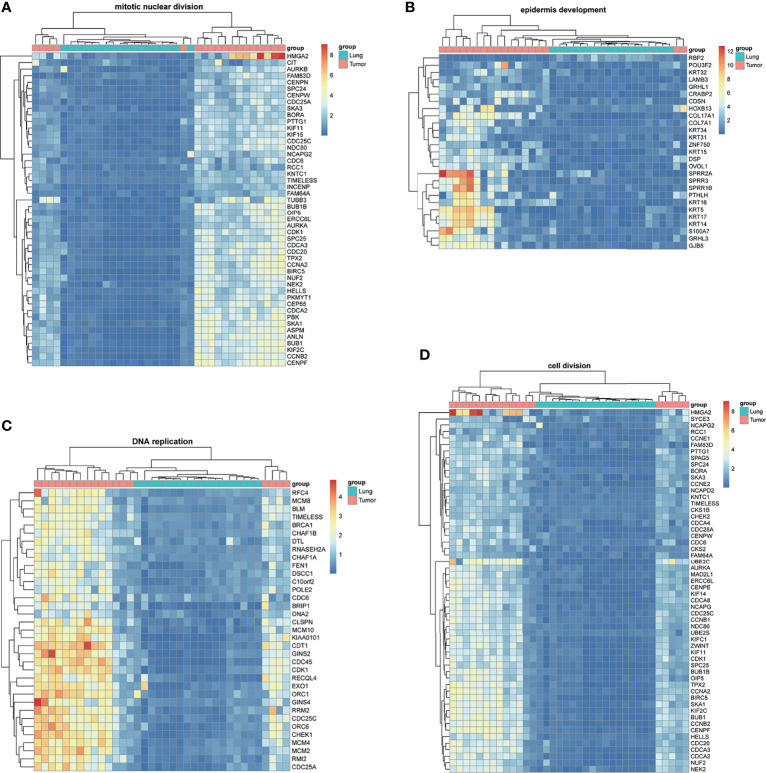
Heatmaps of NSCLC-related differentially expressed genes (DEGs) based on GSE21933. **(A–D)** Hierarchical clustering heatmaps of DEG expression related to sister chromatid cohesion, cell adhesion, leukocyte migration, and cell division.

BM-DEGs and NSCLC-DEGs were confirmed in GSE161116, GSE74706, and GSE21933. In GSE161116, there were 282 DEGs between brain and lung tumor specimens from BM patients, which we defined as BM-DEGs. **Figure 1** shows heatmaps of the expression of the BM-DEGs related to the GO BP terms immune response, inflammatory response, adaptive immune response, and innate immune response. Additionally, in GSE74706, there were 1266 DEGs between lung tumor and normal lung specimens in NSCLC patients and, in GSE21933, there were 2928. We defined these 4194 DEGs as NSCLC-DEGs. **Figure 2** shows heatmaps of the expression of the NSCLC-DEGs in GSE74706 related to the GO BP terms mitotic nuclear division, epidermis development, DNA replication, and cell division. Simultaneously, **Figure 3** shows heatmaps of the expression of the NSCLC-DEGs in GSE21933 related to the GO BP terms sister chromatid cohesion, cell adhesion, leukocyte migration, and cell division.

### GO and KEGG Enrichment Analyses Results

**Figure 4:**
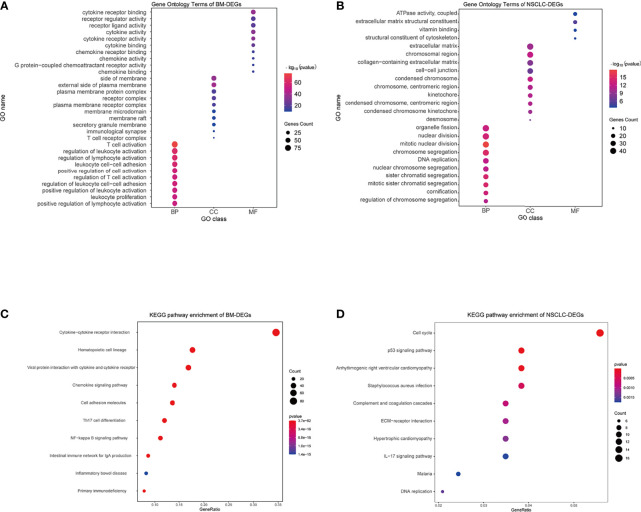
GO and KEGG enrichment results. **(A, B)** GO terms associated with BM- and NSCLC-differentially expressed genes (DEGs). **(C, D)** KEGG pathways associated with BM- and NSCLC-DEGs.

Using the DAVID database, the top five GO BP terms for BM-DEGs were T cell activation, regulation of leukocyte activation, regulation of lymphocyte activation, leukocyte cell–cell adhesion, and positive regulation of cell activation. The top five GO CC terms were cytoplasmic side of membrane, external side of plasma membrane, plasma membrane protein complex, receptor complex, and plasma membrane receptor complex. The top five GO FM terms were cytokine receptor binding, receptor regulator activity, receptor ligand activity, cytokine activity, and cytokine receptor activity (**Figure 4A**). The results indicated that BM-DEGs may promote the occurrence of brain metastasis by mediating the immune responses. Regarding the NSCLC-DEGs, the significantly enriched GO BP terms included organelle fission, nuclear division, mitotic nuclear division, chromosome segregation, and DNA replication. The main CC terms were extracellular matrix, chromosomal region, collagen−containing extracellular matrix, cell−cell junction, and condensed chromosome. Lastly, the MF terms included ATPase activity, extracellular matrix structural constituent, vitamin binding, and structural constituent of cytoskeleton (**Figure 4B**). The results above showed that the NSCLC-DEGs may be related to the occurrence, growth and proliferation of tumors.

Regarding the KEGG pathway analysis, the BM-DEGs were mainly enriched in Cytokine−cytokine receptor interaction, Hematopoietic cell lineage, Viral protein interaction with cytokine and cytokine receptor, Chemokine signaling pathway, and Cell adhesion molecules (**Figure 4C**). The NSCLC-DEGs were mainly enriched in Cell cycle, p53 signaling pathway, and Arrhythmogenic right ventricular cardiomyopathy (**Figure 4D**).

### Protein–Protein Interaction Networks

**Figure 5:**
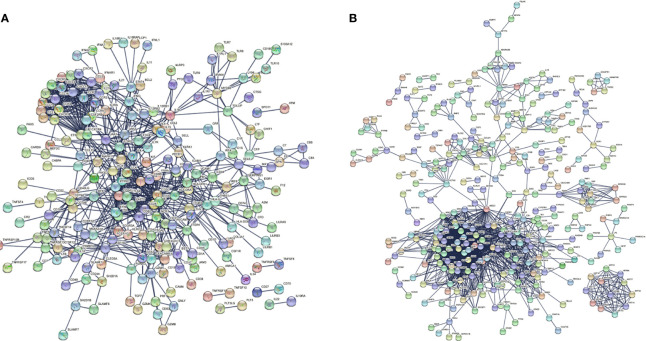
PPI networks of **(A)** BM-differentially expressed genes (DEGs) and **(B)** NSCLC-DEGs using the STRING database.

There were 167 and 245 nodes in the PPI networks of BM- and NSCLC-DEGs, respectively (**Figure 5**). The hub genes in the PPI network of BM-DEGs (related to BM maintenance) included T-cell surface glycoprotein CD3 delta chain (CD3D; degree = 21), T-cell-specific surface glycoprotein CD28 (CD28; degree = 20), T-cell surface glycoprotein CD3 gamma chain (CD3G; degree = 20), Tyrosine-protein kinase Fyn (FYN; degree = 20), C-C motif chemokine 1 (CCL1; degree = 19), C-C motif chemokine 13 (CCL13; degree = 18), T-cell surface glycoprotein CD3 zeta chain (CD247; degree = 18), T-cell surface glycoprotein CD3 epsilon chain (CD3E; degree = 18), T-cell surface glycoprotein CD4 (CD4; degree = 18), and C-C motif chemokine 16 (CCL16; degree = 17) (**Figure 5A** and **Additional File 4**). Further GO enrichment analysis of the biological processes showed that the genes in the most significant module were mainly associated with T cell receptor signaling pathway, antigen receptor-mediated signaling pathway, immune response-activating cell surface receptor signaling pathway, immune response-activating signal transduction, immune response-regulating signaling pathway, and activation of immune response (**Table 2** and **Additional Files 5**).

**Table 2:** Enriched biological processes in the most significant BM-DEG module.

Biological processes	P-value	q-value Bonferroni	Count
T cell receptor signaling pathway	1.34E-08	5.91E-06	219
antigen receptor-mediated signaling pathway	7.68E-08	3.39E-05	338
immune response-activating cell surface receptor signaling pathway	3.64E-07	1.61E-04	498
immune response-activating signal transduction	3.67E-07	1.62E-04	499
immune response-regulating signaling pathway	4.93E-07	2.17E-04	537
activation of immune response	7.54E-07	3.32E-04	597

The hub genes (with a relatively high degree) in the PPI network of NSCLC-DEGs included Centromere protein F (CENPF; degree = 74), G2/mitotic-specific cyclin-B1 (CCNB1; degree = 67), G2/mitotic-specific cyclin-B2 (CCNB2; degree = 61), Cyclin-A2 (CCNA2; degree = 60), Mitotic checkpoint serine/threonine-protein kinase BUB1 (BUB1; degree = 56), and Aurora kinase B (AURKB; degree = 56) (**Figure 5B** and **Additional File 6**). Further GO enrichment analysis of the biological processes showed that the genes in the most significant module were mainly associated with spindle assembly checkpoint signaling, mitotic spindle assembly checkpoint signaling, mitotic spindle checkpoint signaling, negative regulation of mitotic metaphase/anaphase transition, negative regulation of metaphase/anaphase transition of cell cycle, and negative regulation of mitotic sister chromatid separation (**Table 3** and **Additional File 7**).

**Table 3:** Enriched biological processes in the most significant NSCLC-DEG module.

Biological processes	P-value	q-value Bonferroni	Count
spindle assembly checkpoint signaling	3.09E-08	1.34E-05	41
mitotic spindle assembly checkpoint signaling	3.09E-08	1.34E-05	41
mitotic spindle checkpoint signaling	3.58E-08	1.55E-05	43
negative regulation of mitotic metaphase/anaphase transition	3.58E-08	1.55E-05	43
negative regulation of metaphase/anaphase transition of cell cycle	4.12E-08	1.78E-05	45
negative regulation of mitotic sister chromatid separation	4.41E-08	1.91E-05	46

### Functional Enrichment of co-DEGs

**Figure 6:**
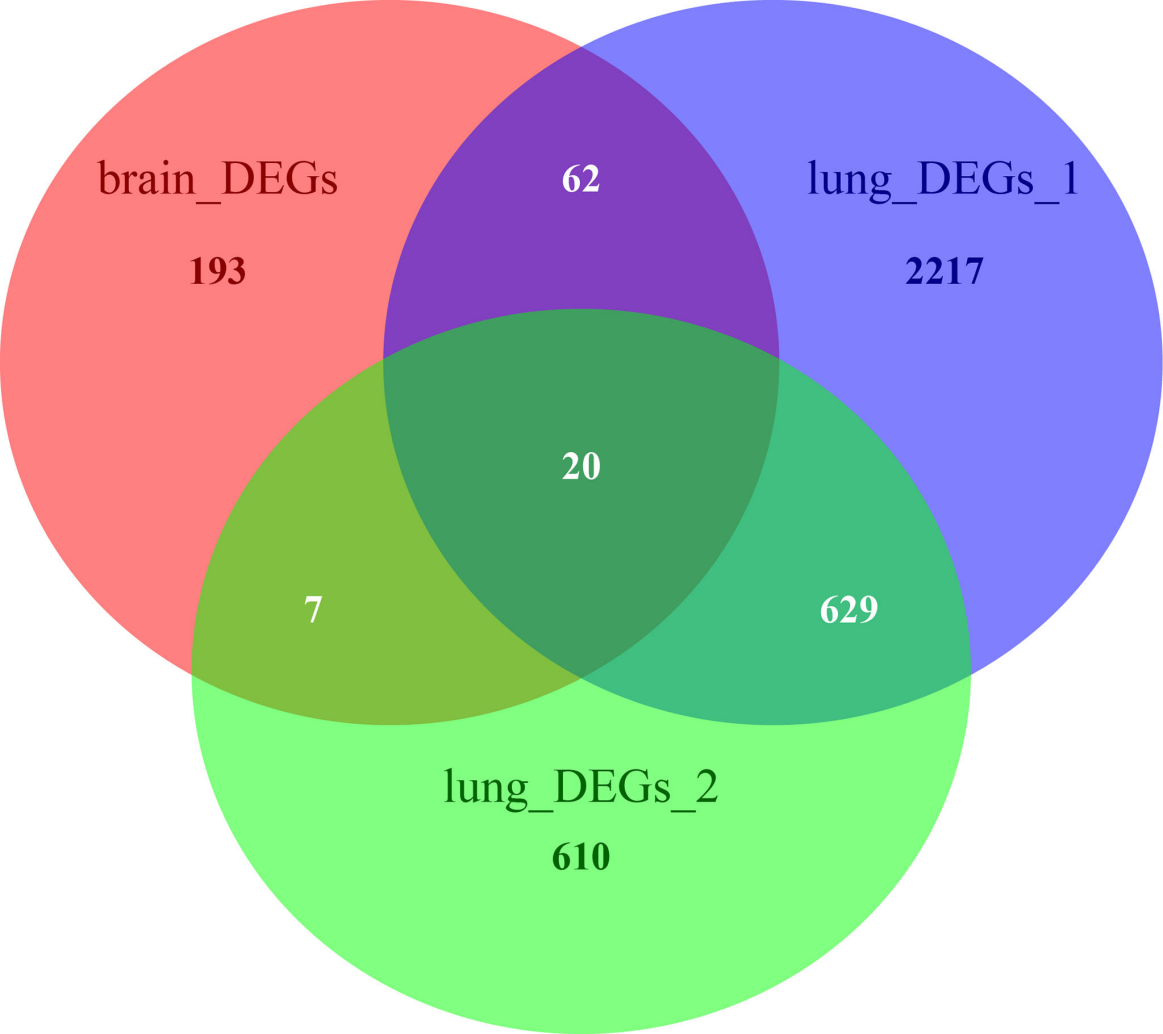
Venn diagrams of BM- and NSCLC-differentially expressed genes (DEGs), 20 of which were co-DEGs (shared DEGs).

The Venn diagram in **Figure 6** illustrates the BM- and NSCLC-DEGs, revealing 20 co-DEGs, which were identified as macrophage receptor (MARCO), leucine-rich repeat neuronal protein 3 (LRRN3), complement factor D (CFD), cadherin-5 (CDH5), GTP-binding protein RAD (RRAD), platelet endothelial cell adhesion molecule 1 (PECAM-1), complement component C8 beta chain (C8B), lysosomal-associated membrane protein 3 (LAMP3), deleted in malignant brain tumors 1 protein (DMBT1), interleukin-7 receptor subunit alpha (IL7R), early growth response protein 1 (EGR1), complement component C7 (C7), interleukin-6 (IL6), B-lymphocyte antigen CD19 (CD19), coagulation factor XII (F12), interleukin-11 (IL11), C4b-binding protein alpha chain (C4BPA), C-X-C motif chemokine 13 (CXCL13), E-selectin (SELE), and C-C motif chemokine 2 (CCL2). The GO enrichment and KEGG pathway analysis results are shown in **Additional Files 8**, **9**.

### Predicted TFBSs That May Regulate the co-DEGs

Prediction analysis using ToppGene bioinformatics tools identified the TFBSs that regulate the 20 co-DEGs (**Table 4**). Among the 6 TFBSs, nuclear factor of activated T cells (NFAT) was identified among the DEGs of all three datasets at the same time (**Additional Files 10** **–** **12**). The results showed that NFAT could modulate the expression of IL-11, CDH5, CCL2, IL-6, LRRN3, and IL7R. Among NFAT isoforms, NFATc1 and NFATc3 are meaningfully downregulated among NSCLC-DEGs (p < 0.05). Among the BM-DEGs, NFATc1, NFATc2, NFATc3, and NFATc4 were significantly downregulated (p < 0.05). Therefore, NFATc1 and NFATc3 may play key roles in the occurrence of both NSCLC and BM.

**Table 4:** Transcription factor binding site of the 20 co-DEGs.

Transcription factor	Sequence	P-value	Hit Count in Query List	Hit in Query List
ETS2	RYTTCCTG_V$ETS2_B	1.21E-06	7	SELE, CD19, EGR1, CDH5, CCL2, MARCO, IL7R
OLF1	V$OLF1_01	1.44E-05	4	CD19, IL11, EGR1, LRRN3
AP1	TGANTCA_V$AP1_C	3.47E-04	5	IL11, CDH5, IL6, LRRN3, IL7R
NFAT	TGGAAA_V$NFAT_Q4_01	4.09E-04	6	IL11, CDH5, CCL2, IL6, LRRN3, IL7R
AP4	CAGCTG_V$AP4_Q5	1.31E-03	5	IL11, EGR1, LRRN3, C7, RRAD

### Survival Analysis of IL-11, CDH5, CCL2, IL-6, LRRN3, and IL-7R

**Figure 7:**
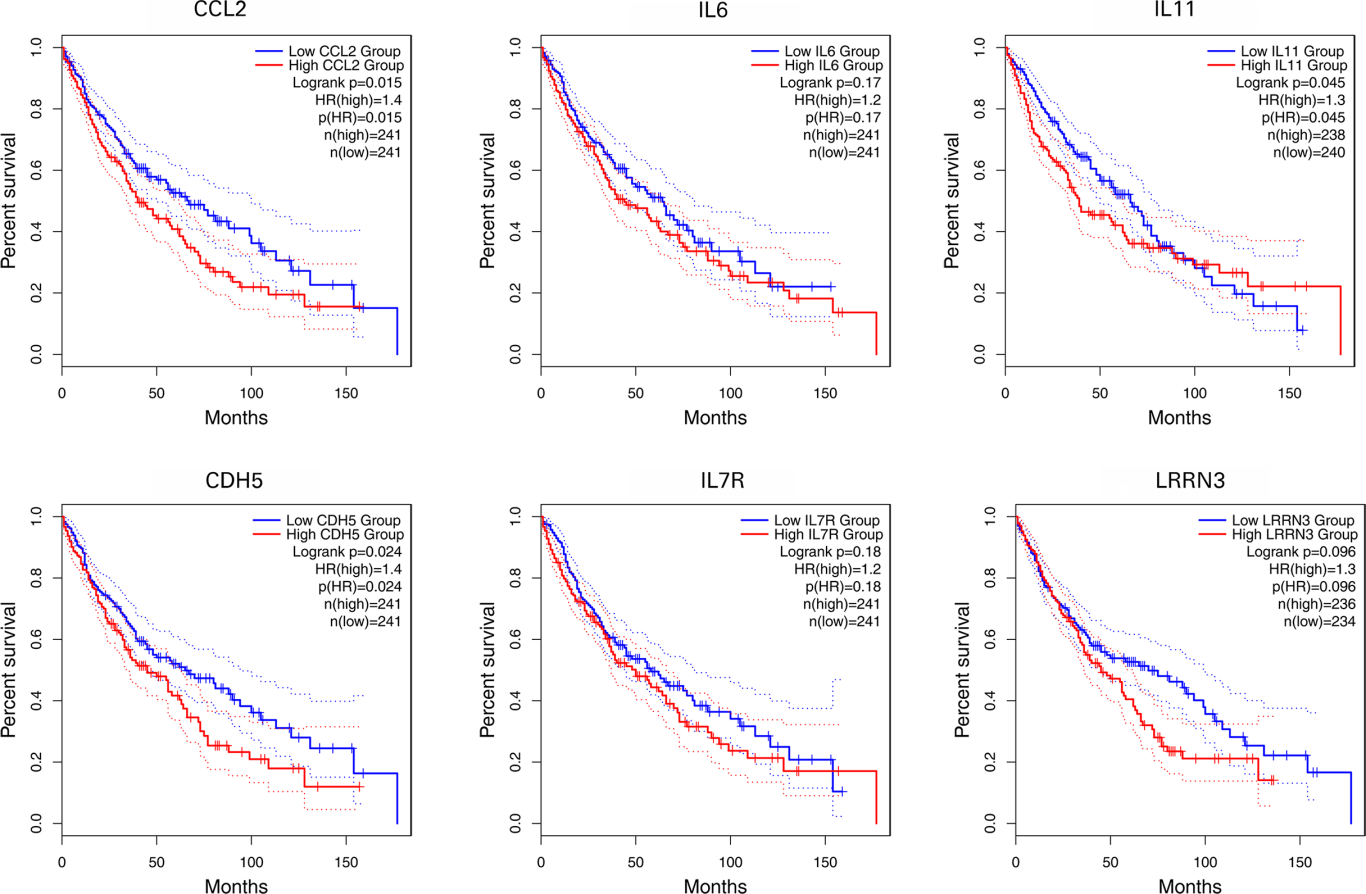
Survival analyses of IL11, CDH5, CCL2, IL6, LRRN3, and IL7R.

Using primarily the datasets from TCGA and GEO, the expression of 6 NFAT-regulated co-DEGs was correlated with the prognosis of patients with NSCLC. As shown in **Figure 7**, the highly expressed IL-11, CDH5, and CCL2 were linked with poor prognosis based on overall survival among patients with NSCLC (p < 0.05).

### Immunohistochemistry

To validate the predictive results, tissue samples of BMs, lung cancer, and matched neighboring normal lung were obtained from 30 patients with BMs due to NSCLC who underwent surgical resection at the Second Affiliated Hospital of Chongqing Medical University, China (September 2016 to June 2021).

**Figure 8:**
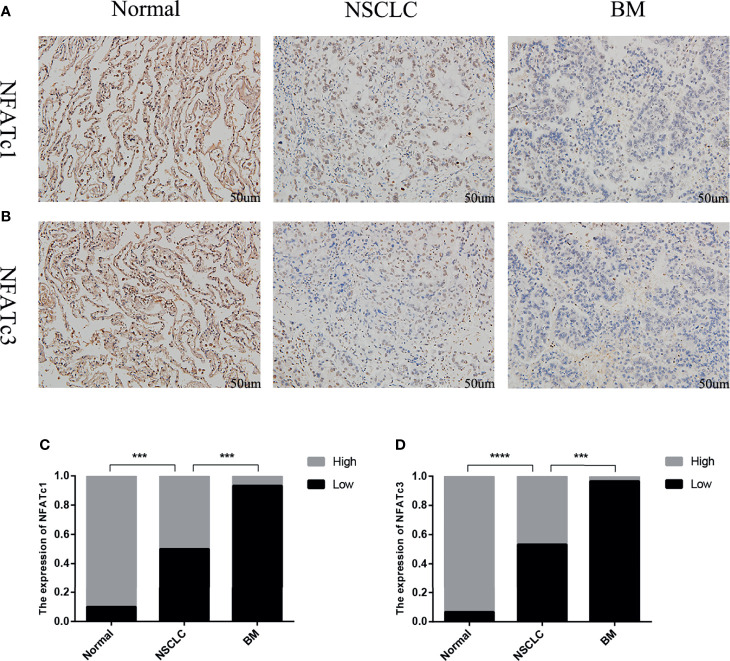
Expression of NFATc1 and NFATc3 in normal lung tissue, NSCLC and BM. **(A)** Immunohistochemical analysis of normal tissues, NSCLC and BM with NFATc1 expression (Scale bars: 50 μm). **(B)** Immunohistochemical analysis of normal tissues, NSCLC and BM with NFATc3 expression (Scale bars: 50 μm). **(C, D)** The expression of NFATc1 **(C)**, and NFATc3 **(D)** cells in normal tissues, NSCLC and BM was quantified. Data represent the proportion of each category. ***P < 0.001, ****P < 0.0001.

The immunohistochemistry results showed that the expression levels of NFATc1 and NFATc3 in NSCLC were dramatically lower (**Figure 8**) than those in matched normal tissues. In BM, the expression levels of NFATc1 and NFATc3 were significantly lower than those in the matched NSCLC tissues (**Figure 8**). These results are highly consistent with the results of the bioinformatics analysis.

## Discussion

Approximately 25% of NSCLC patients develop BM, which is associated with poor prognosis and significant mortality and can cause neurologic, cognitive, and emotional difficulties. To mitigate morbidity and mortality, patients at risk of BM must be identified. However, efforts to characterize NSCLC patients who develop BM have been fairly disappointing. Identifying potential biomarkers and associations that link BM and NSCLC are thus of interest and may represent novel therapeutic targets.

The bioinformatics analysis showed that the processes associated with NSCLC are primarily related to cell proliferation and differentiation, whereas the processes associated with BM are related to immune and inflammatory reactions.

Most importantly, NFAT may play an important role in the process through which NSCLC develops into BM by regulating IL-11, CDH5, CCL2, IL-6, LRRN3, and IL-7R. IL-11 is a member of the family of glycoprotein-130 (GP-130) cytokines, which utilizes the GP-130 signaling pathway (19). IL-11 activates the Janus kinase (JAK)–signal transducer and activator of transcription 3 (STAT3) pathway (20, 21). We observed similar results, as demonstrated in **Additional File 6**. Many tumor-promoting effects of IL-11, such as the promotion of cancer cell migration and invasion, have been linked to the STAT3 signaling cascade (22, 23). The GP-130–dependent JAK–STAT3 pathway induces matrix metalloproteinases (MMPs) 2, 7, and 9, which play roles in the degradation of the extracellular matrix and facilitate the epithelial–mesenchymal transition (EMT) (24–26). The EMT has been considered an important mechanism for cancer cell invasion and distant metastasis formation (21). We believe that IL-11 may promote the occurrence of BMs from NSCLC by activating these processes. CDH5, also known as vascular endothelial CDH, is a member of the transmembrane cadherin superfamily, generally considered to play key roles in the progression of various malignant tumors (27). CDH5 plays important roles in cell adhesion, contact inhibition of growth, and the inhibition of endothelial cell migration and apoptosis (28). In glioma, CDH5 overexpression contributed to the vasculogenic mimicry of glioblastoma stem-like cells and was found to be an independent adverse prognostic predictor for glioblastoma multiforme patients (29). In breast carcinoma, CDH5 expression is upregulated and serves as a metastasis marker (30). In NSCLC, the increased expression of CDH5 was associated with increased angiogenesis in lung cancer cells, promoting the migration and invasion of lung cancer cells (31). Chemokines are a superfamily of secreted proteins involved in inflammatory and immunoregulatory processes, and CCL2 has been implicated in the pathogenesis of various disease processes. Significantly high CCL2 expression levels have been detected in the epithelial regions of many tumor types (32–34). Furthermore, in BM tumors, the increased secretion of CCL2 recruits Iba1^+^ myeloid cells, which reciprocally enhance BM tumor cell outgrowth *via* enhanced proliferation and reduced apoptosis (35, 36).

The NFAT family of TFs is comprised of five members, four of which, NFATc1–NFATc4, are regulated by Ca^2+^-calcineurin signaling. NAFTc1 and NFATc2 are the primary NFAT isoforms expressed in T cells and play key roles in the regulation of early gene transcription in response to T cell receptor–mediated signals (37). A recent study found that NFATc1-deficient cytotoxic T cells showed reduced cytotoxicity against tumor cells (38). In addition, the expression of programmed cell death protein 1 (PD-1), one of the most successfully targeted checkpoint proteins across various cancer types, including NSCLC, is induced by NFATc1 following T cell activation (39). A recent study showed that the targeted deletion of NFATc1 in T cells increased lung tumor growth in mice, related to T cell activation and impaired function. In human patients, the downregulation of NFATc1 in T cells has been identified in NSCLC and advanced disease stages, indicating that the absence of NFATc1 in T cells is associated with poor prognosis (40). Research on NFATc3 in BM due to NSCLC remains lacking. However, NFATc3 is known to play an important role in the control of neuronal survival. A recent study demonstrated that the overexpression of NFATc3 aggravated neuronal death, whereas the knockdown of NFATc3 protected neurons from apoptosis (41). In hepatocellular carcinoma, NFATc3 is frequently deleted or downregulated, which is associated with a poor prognosis among hepatocellular carcinoma patients (42). In T-cell lymphomas, NFATc3 has been identified as a tumor suppressor (43). Our research identified the downregulation of NFATc3 in NSCLC and BM, suggesting that NFATc3 could represent a new biomarker and therapeutic target for NSCLC-related BM.

## Conclusion

In summary, NFATc1 and NFATc3 may play important roles in the occurrence of NSCLC and BM by regulating IL-11, CDH5, and CCL2. NFATc1 and NFATc3 may serve as biomarkers for the characterization of NSCLC patients at risk for BM, and NFATc1 and NFATc3 might serve as targets for the treatment of NSCLC.

## Data Availability Statement

Publicly available datasets were analyzed in this study. This data can be found here: http://www.ncbi.nlm.nih.gov/geo/GSE161116 GSE74706 GSE21933.

## Ethics Statement

The studies involving human participants were reviewed and approved by Institutional Review Board of Chongqing Medical University (2021 Scientific Ethics Approval No. 68). The patients/participants provided their written informed consent to participate in this study. Written informed consent was obtained from the individual(s) for the publication of any potentially identifiable images or data included in this article.

## Author Contributions 

LP takes responsibility for all aspects of the reliability and freedom from bias of the data presented and their discussed interpretation, drafting the article. YT, RW, JS, and MS collected data. YC and ZX analysed the results. JM, XZ, and GL take responsibility for full text evaluation and guidance. All authors contributed to the article and approved the submitted version.

## Funding

This study was supported by National Nature Science Foundation of People’s Republic of China (81401500), Science Foundation of Chongqing (cstc2019jcyj-msxmX0231), Science Foundation of Jiangsu Province, People’s Republic of China (BK20140298). High-level Medical Reserved Personnel Training Project of Chongqing. Kuanren Talents Program of the second affiliated hospital of Chongqing Medical University and Chongqing Scientific and Health Joint Medical Research Project (2020GDRC021).

## Conflict of Interest

The authors declare that the research was conducted in the absence of any commercial or financial relationships that could be construed as a potential conflict of interest.

## Publisher’s Note

All claims expressed in this article are solely those of the authors and do not necessarily represent those of their affiliated organizations, or those of the publisher, the editors and the reviewers. Any product that may be evaluated in this article, or claim that may be made by its manufacturer, is not guaranteed or endorsed by the publisher.

## Glossary

BMs: brain metastases
NSCLC: non-small cell lung cancer
PCI: prophylactic cranial irradiation
DEG: differentially expressed gene
co-DEG: co-differentially expressed gene
TFBS: Transcription Factor Binding Site
NSCLC-DEG: NSCLC-related DEG
BM-DEG: BM-related DEG
ADC: adenocarcinoma
SCC: squamous cell carcinoma
FDR: false discovery rate
FC: fold change
PPI: protein–protein interaction
GO: Gene Ontology
KEGG: Kyoto Encyclopedia of Genes and Genomes
MARCO: macrophage receptor
LRRN3: leucine-rich repeat neuronal protein 3
CFD: complement factor D
CDH5: cadherin-5
RRAD: GTP-binding protein RAD
PECAM-1: platelet endothelial cell adhesion molecule
C8B: complement component C8 beta chain
LAMP3: lysosomal-associated membrane protein 3
DMBT1: deleted in malignant brain tumors 1 protein
IL7R: interleukin-7 receptor subunit alpha
EGR1: early growth response protein 1
C7: complement component C7
IL6: interleukin-6
CD19: B-lymphocyte antigen CD19
F12: coagulation factor XII
IL11: interleukin-11
C4BPA: C4b-binding protein alpha chain
CXCL13: C-X-C motif chemokine 13
SELE: E-selectin
CCL2: C-C motif chemokine 2
BP: biological process
CC: cellular component
MF: molecular function
TFs: Transcription Factors
TFBS: Transcription Factor Binding Site
GEPIA: Gene Expression Profiling Interactive Analysis
NFAT: Nuclear factor of activated T-cells
IL-11: Interleukin-11
TCGA: The Cancer Genome Atlas
HNSC: head and neck squamous cell carcinoma
LUAD: lung adenocarcinoma
LGG: brain lower-grade glioma
TAM: tumor-associated macrophage
miRNA: microRNA
GP-130: glycoprotein-130
JAK: Janus kinase
STAT3: signal transducer and activator of transcription 3
MMP: matrix metalloproteinase
EMT: epithelial-mesenchymal transition
IL-7: Interleukin-7
