# Supplementary figures and images for: NFAT as a Biomarker and Therapeutic Target in Non–Small Cell Lung Cancer–Related Brain Metastasis
Source: Front Oncol. 2021 Nov 30;11:781150. doi: 10.3389/fonc.2021.781150 (PMC8669620; doi:10.3389/fonc.2021.781150)

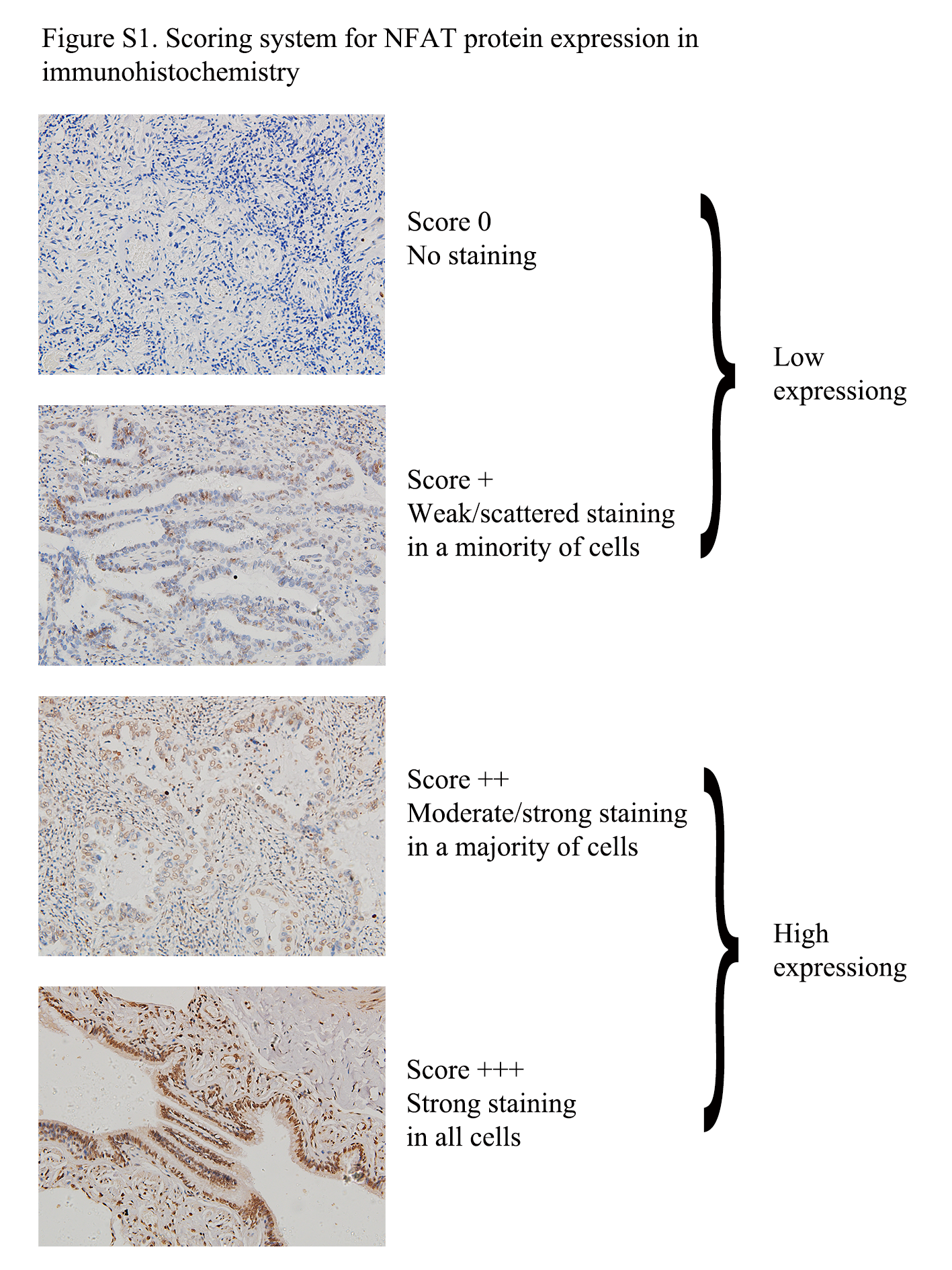

Supplement: Supplementary file 10 [file Image_1.tif]

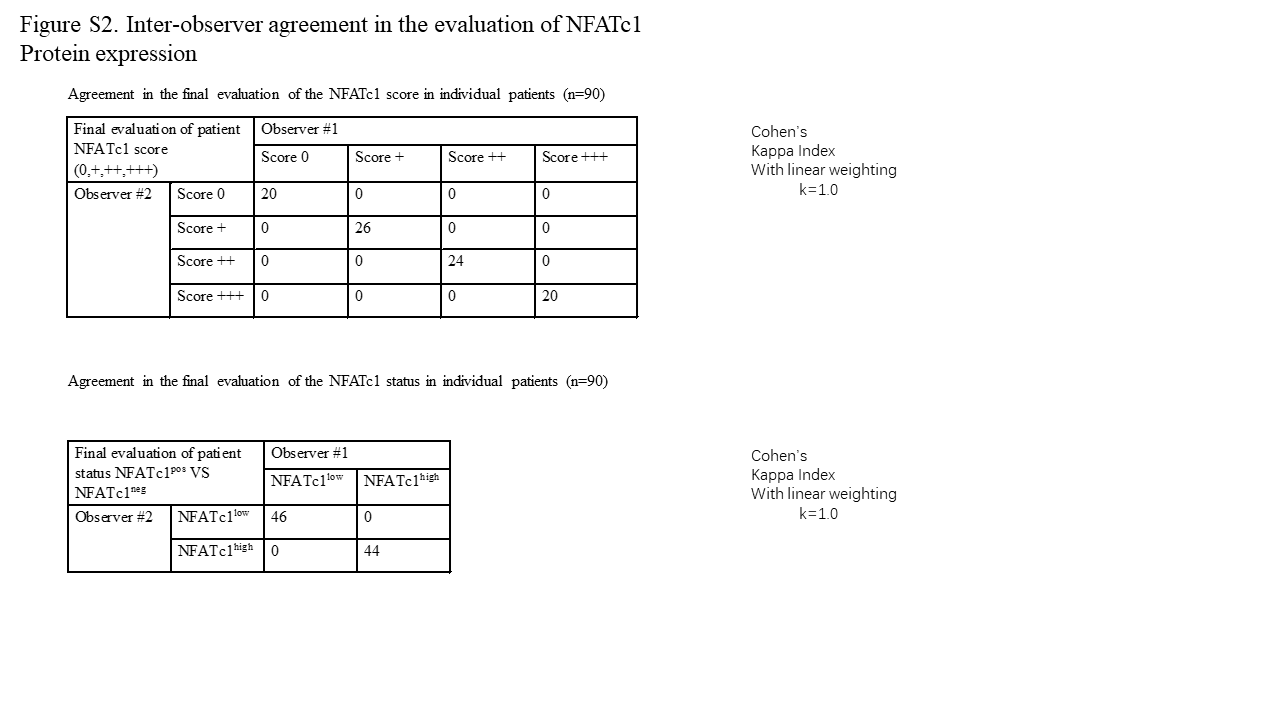

Supplement: Supplementary file 11 [file Image_2.tif]

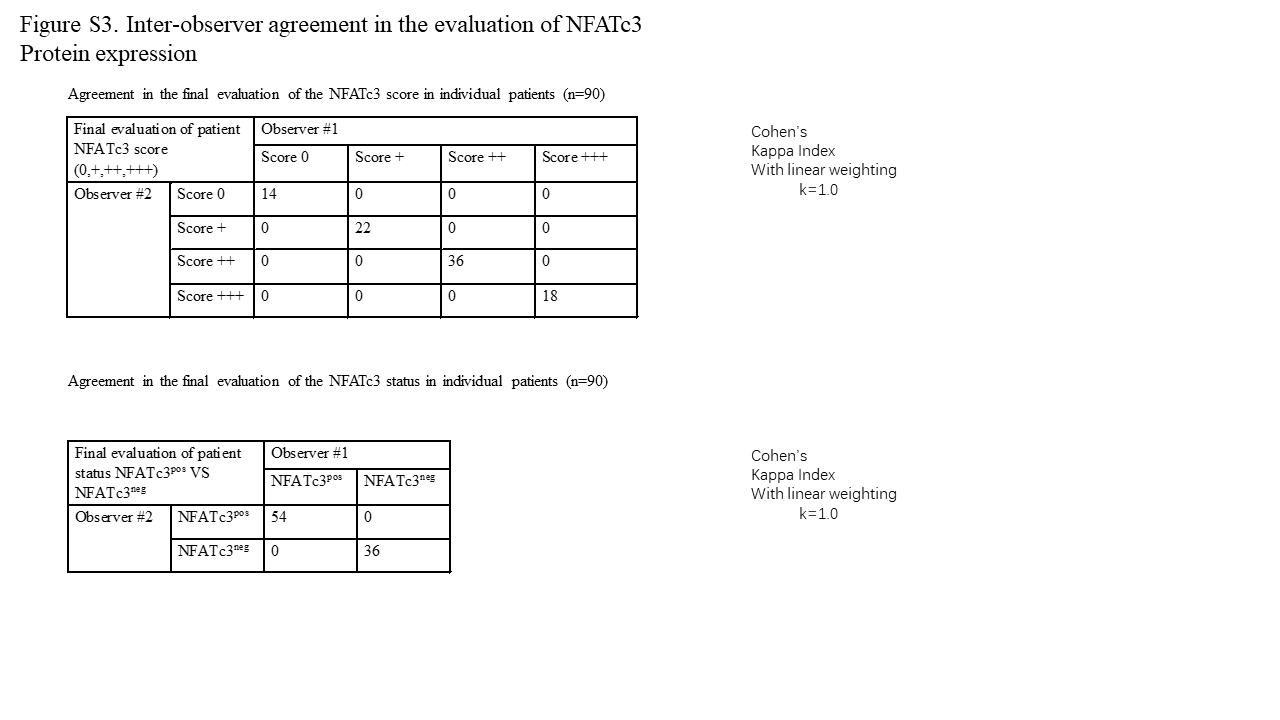

Supplement: Supplementary file 12 [file Image_3.tif]
